# Development of a disease-specific quality of life questionnaire for adult patients with hereditary angioedema due to C1 inhibitor deficiency (HAE-QoL): Spanish multi-centre research project

**DOI:** 10.1186/1477-7525-10-82

**Published:** 2012-07-20

**Authors:** Nieves Prior, Eduardo Remor, Carmen Gómez-Traseira, Concepción López-Serrano, Rosario Cabañas, Javier Contreras, Ángel Campos, Victoria Cardona, Stefan Cimbollek, Teresa González-Quevedo, Mar Guilarte, Dolores Hernández Fernández de Rojas, Carmen Marcos, María Rubio, Miguel Ángel Tejedor-Alonso, Teresa Caballero

**Affiliations:** 1 Allergy Department, IdiPaz (Hospital La Paz Health Research Institute), Madrid, Spain; 2 Universidad Autónoma de Madrid, Madrid, Spain; 3 Hospital Universitario La Fe, Valencia, Spain; 4 Hospital Universitario Vall d’Hebron, Barcelona, Spain; 5 Hospital Universitario Virgen del Rocío, Sevilla, Spain; 6 Hospital Xeral Cíes, Vigo, Spain; 7 Hospital General Universitario Gregorio Marañón, Madrid, Spain; 8 Allergy Unit, Hospital Universitario FundaciÃ³n AlcorcÃ³n, Madrid, Spain; 9 Biomedical Research Network on Rare Diseases U754 (CIBERER), Madrid, Spain

**Keywords:** Hereditary angioedema, C1 inhibitor deficiency, Quality of Life, Questionnaire, Qualitative methodology

## Abstract

**Background:**

There is a need for a disease-specific instrument for assessing health-related quality of life in adults with hereditary angioedema due to C1 inhibitor deficiency, a rare, disabling and life-threatening disease. In this paper we report the protocol for the development and validation of a specific questionnaire, with details on the results of the process of item generation, domain selection, and the expert and patient rating phase.

**Methods/Design:**

Semi-structured interviews were completed by 45 patients with hereditary angioedema and 8 experts from 8 regions in Spain. A qualitative content analysis of the responses was carried out. Issues raised by respondents were grouped into categories. Content analysis identified 240 different responses, which were grouped into 10 conceptual domains. Sixty- four items were generated. A total of 8 experts and 16 patients assessed the items for clarity, relevance to the disease, and correct dimension assignment. The preliminary version of the specific health-related quality of life questionnaire for hereditary angioedema (HAE-QoL v 1.1) contained 44 items grouped into 9 domains.

**Discussion:**

To the best of our knowledge, this is the first multi-centre research project that aims to develop a specific health-related quality of life questionnaire for adult patients with hereditary angioedema due to C1 inhibitor deficiency. A preliminary version of the specific HAE-QoL questionnaire was obtained. The qualitative analysis of interviews together with the expert and patient rating phase helped to ensure content validity. A pilot study will be performed to assess the psychometric properties of the questionnaire and to decide on the final version.

## Background

Hereditary angioedema due to C1 inhibitor deficiency (HAE-C1-INH) is a genetic disease that reduces the synthesis or function of C1 inhibitor (C1-INH) [1]. Bradykinin acts as the primary mediator [2]. It is considered a rare disease, and in Spain has a minimal prevalence rate of 1.09 cases/100,000 inhabitants [3]. HAE-C1-INH is characterized by recurrent edema attacks affecting different parts of the body (e.g.: face, extremities, gastrointestinal track, upper airway) [1,4]. The clinical expression is quite variable; patients may be asymptomatic or suffer from life-threatening angioedema episodes with a wide range of disabling symptoms, severe pain or disfigurement. Attacks are unpredictable. Abdominal attacks may mimic acute abdominal emergencies, prompting unnecessary surgery. Mortality is mainly due to upper airway obstruction caused by laryngeal edema and has been reported to be as high as 30-50% in patients with undiagnosed HAE-C1-INH [4] and 30% in patients diagnosed with HAE-C1-INH whose cases were improperly treated [5].

Conventional treatment for allergic angioedema with adrenaline, antihistamines and glucocorticoids has not been shown efficacious [6].

Therapeutic management of HAE-C1-INH focuses on either preventing or treating attacks. Although new drugs have been developed in recent years, their availability differs from country to country and may even vary among hospitals within a country. Attenuated androgens, antifibrynolytics and plasma derived human C1 inhibitor concentrate (pdhC1INH) are the most commonly used drugs for long term prophylactic treatment. Election treatment for acute episodes are replacement of the lacking protein (pdhC1INH, nanofiltered pdhC1INH or recombinant C1INH concentrate), icatibant acetate or ecallantide and fresh frozen plasma when the aforementioned treatments are not available [7]. Different consensus guidelines for management of HAE-C1-INH have been published [7-13]. Several drug adverse effects and contraindications have been reported (see Table 1). 

**Table 1:** Secondary adverse events and contraindications of treatments in HAE-C1-INH

**Drug**	**Secondary Adverse Events**	**Contraindications**
**Attenuated androgens**	Virilization, headache, depression, weight gain, menstrual irregularities, sleeplessness, agitation, mood changes, arterial hypertension, dyslipemia, decreased libido and hepatotoxiciy [14-17].	Pregnancy, lactation, childhood, breast cancer, prostate carcinoma, nephrotic syndrome, significant alteration of hepatic function [7].
**Antifibrinolytics**	Nausea, vomiting, headache, diarrhea, orthostatic regulation disturbances, myositis, muscle necrosis, increase in the risk of thrombosis [1].	History of thrombosis or thromboembolism [7].
**Fresh frozen plasma**	Transmission of infectious diseases, potential aggravation of edema symptoms due to substrate supply that may lead to an increase of bradykinin, alloimmunization, anaphylactic or allergic reactions, excessive intravascular volume with risk of hypervolemia and heart failure [7].	
**pdhC1INH**	Potential transmission of infectious and/or pathogenic viruses [18], infection at injection site and thrombosis associated with indwelling catheters used for the administration of long-term prophylaxis with pdhC1inh concentrate [12].	
**Icatibant acetate**	Minor local adverse effects (self-limited erythema, pruritus and pain in injection area) [19,20].	Active ischemic heart diseaseIctus in previous 2 weeks [20].
**Ecallantide**	Anaphylactic and other acute allergic reactions [21]	
**Recombinant human C1INH**	Allergic reaction [22].	Rabbit allergy [7].

The unavailability of some HAE-C1-INH specific therapies, as well as the potential side effects and contraindications of treatments could significantly hinder the management of HAE-C1-INH patients. Moreover, the lack of awareness of HAE-C1-INH among health care professionals may cause patients to fear for their safety and could have a negative effect on health-related Quality of Life (HRQoL).

HRQoL is considered to be a subjective assessment of the impact of disease and treatment across physical, psychological, social and somatic domains of functioning and well-being [23]. Several aspects of HAE-C1-INH (hereditary transmission, improper diagnosis, unpredictability of attacks, disabling symptoms, risk of fatal attacks, unnecessary surgeries, inadequate treatments, frequent need for emergency intervention, lack of information about the disease among health care professionals, adverse effects of treatment or unavailability of specific treatment, etc.) could diminish HRQoL, and the impact of HAE-C1-INH on quality of life should be evaluated. However, there is limited data on this topic in the published literature [24-28]. To the best of our knowledge, a specific instrument for assessing the impact of HAE-C1-INH on HRQoL has not yet been made available.

This HAE-QoL project aims to develop a sound psychometric tool with which to assess HRQoL in HAE-C1-INH. It has been designed with two phases: the first was for developing the draft version of the questionnaire and the remaining phase will be for assessing its psychometric characteristics. Once the instrument has been validated, we will perform a HRQoL study using the final version of the questionnaire.

The results derived from the first phase of the study, in which domains were chosen and items were identified for the first draft of the HAE-QoL questionnaire, are described in this manuscript. We have also included the results of the expert and patient rating phase in which we received feedback for assessing both content and face validity. Lastly, this paper contains the protocol for the future pilot study and psychometric analysis.

## Methods and design

Ethical approval was granted by the Research Ethics Committee of Hospital Universitario La Paz (Madrid). HAE-C1-INH patients 18 years or older and HAE-C1-INH experts from different regions of Spain participated in the study. Inclusion criteria for patients, were that they be 18 years old or older and have a confirmed laboratory diagnosis of HAE-C1-INH (type I or II). Exclusion criteria were cognitive disabilities and lack of fluency in Spanish language.

Participating patients signed an informed consent form. An in-house scoring system for classifying the severity of HAE-C1-INH (asymptomatic, mild, moderate and severe) in patients was established by taking into account the frequency of symptoms, the occurrence of life-threatening angioedema attacks and the need for maintenance therapy (see Table 2). For this step, a convenience sample was formed with patients with different degrees of disease severity. A main priority was to include subjects with the most severe clinical expression since they were the ones who theoretically would suffer a greater burden on their QoL.

**Table 2:** Severity Score (over the last year)

**Severity Score**	**Criteria**
**ASYMPTOMATIC**	No angioedema episodes and no long term prophylactic treatment.
**MILD**	No life-threatening angioedema episodes, no long term prophylactic treatment and ≤ 6 episodes/year.
**MODERATE**	No life-threatening angioedema episodes and ≤ 12 episodes/year with long term prophylactic treatment (exclude maintenance treatment with pdhC1INH) or > 6 episodes/year without long term prophylactic treatment.
**SEVERE**	Life-threatening angioedema episode and/or > 12 episodes/year with long term prophylactic treatment and/or maintenance treatment with pdhC1INH.

The questionnaire was developed according to standard questionnaire development guidelines and methodologies, as well as the protocol for development of a specific HRQoL questionnaire for hemophilia, a disease that has similar characteristics to HAE-C1-INH [29-36]. A patient-centred perspective based on a qualitative methodology was chosen in order to ensure that the content was appropiate and relevant for the target population and did not omit any issues of importance to the HAE-C1-INH patient.

Semi-structured individual interviews of HAE-C1-INH patients and experts were conducted using a written self-administered questionnaire. Subjects were asked 8 questions about their personal views on the disease (Table 3). Answers were transcribed and a qualitative content analysis of the responses was carried out according to published methodology [33,34], in order to build a theoretical model. The procedure followed an inductive approach whereby transcripts were encoded in order to develop conceptual categories (domains), in which to classify issues that appeared in the data. Identified responses were organized in terms of frequency and issue, and common issues were grouped into domains. Lastly, items were generated using the reports on the verbatims from patients and experts. Every procedure was double checked by at least two members of the research team in order to ensure accuracy. 

**Table 3:** Qualitative interviewing: semi-structured questions answered in interviews with HAE-C1-INH patients and experts

**Questions for HAE-C1-INH patients**	
1	Without using medical or technical terms, how would you describe your disease?
2	Name the five aspects of your life that are most important to you.
1	Of the aspects you have mentioned, which one is the most affected by your disease at this time?
2	What bothers you most about your disease?
3	What worries you most about your disease?
4	What has made you feel upset, pensive or annoyed in relation to your disease?
5	In what ways does your disease limit you?
6	Is there anything else that you would like to add about your disease?
**Questions for HAE-C1-INH experts:**	
1	Without using medical or technical terms, how would you describe HAE-C1-INH?
2	Name the five aspects of life that in your opinion are the most important for a HAE-C1-INH patient.
3	Of the aspects you have mentioned, which one is the most affected by HAE-C1-INH?
4	In your opinion, what aspect of the disease most bothers a HAE-C1-INH patient?
5	What do you think most worries a HAE-C1-INH patient about his/her disease?
6	What do you think makes a HAE-C1-INH patient feel upset, pensive or annoyed in relation with his/her disease?
7	In what ways does the disease limit a HAE-C1-INH patient?
8	Is there anything else that you would like to add about HAE-C1-INH?

Our HAE-C1-INH patient group (29 females and 16 males) had a mean age of 39 years (18–74 years) and different degrees of HAE-C1-INH severity. These patients and the 8 HAE-C1-INH experts who participated in the study came from 8 regions of Spain (Madrid, Andalusia, Catalonia, Galicia, Asturias, the Valencian Community, Castille and Leon, and the Basque Country).

A content analysis of the interviews identified 240 different responses (*verbatims*). Subjects referred to relationships with health care professionals, work, studies, family, leisure activities, sport, travel, aesthetics, emotional life, sex life, fear of a fatal episode, death of relatives, and treatment (side effects, availability, and new therapies).

The most representative verbatim reports were used to formulate questions for developing an optimal item pool (examples are shown in Table 4). Content analysis led to the creation of 10 conceptual categories or domains: health conditions, physical functioning, social role, emotional role, physical role, symptoms, general health, aesthetics, mental health and treatment. A set of 64 items was developed and they were grouped into these domains. A theoretical model of the questionnaire was built based on these domains and served as the basis for the draft version of the HRQoL questionnaire (HAE-QoL v 1.0). A 5 or 6-point Likert scale (or summative scale) will be used.

**Table 4:** **Examples of significant units (*verbatims*) and items**

**Significant units (*Verbatims*)**	**Domains**	Item*
“Few doctors know about my disease”	Health conditions	How often have you felt that health professionals do not know about your disease?
“I am worried about transmitting the disease to my children”	Emotional role	How much do you worry about the transmission of the disease to your children?
“I feel insecure when traveling, due to the unavailability of C1 inhibitor concentrate”	Social role	How has your disease limited your traveling?
“The truth is that I am always frightened”	Mental Health	To what extent does your disease make you feel anxious or fearful?
“I am worried about the adverse side effects of medication”	Treatment	How much are you worried about the adverse side effects of treatment?
“An important aspect that is affected is work. Attacks are more frequent at exam time”	Physical role	How has your disease affected your work or studies during the last six months?
“Barrier to regulating menstruation”	General Health	Have you had problems in receiving adequate treatment for other diseases (drugs for arterial hypertension, oral contraceptives, and other hormonal treatments) due to hereditary angioedema?
“When I have an attack, I cannot walk, wash or groom myself”.	Physical functioning	Have your angioedema episodes prevented you from accomplishing basic activities of daily life (washing, eating, walking, etc. ?
“I had to give up medication because of virilization symptoms”	Aesthetics	Have you been affected by the possibility of suffering changes in your physical appearance due to the side effects of attenuated androgens?

Note: *Verbatim reports and items were translated from original Spanish into English for this article.

The definitions of the preliminary domains of the questionnaire and the number of items per domain in the draft version are shown in Table 5.

**Table 5:** Category definitions and number of items per category in HAE − QoL questionnaire

**Domain**	**Definition**	**N° of items**	**N° of items**
		**HAE-QOL v 1.0**	**HAE-QOL v 1.1**
**Health conditions**	To what extent the availability of adequate assistance from health services and the information (related to prevention and treatment) offered by health personnel affects daily life.	11	4
**Physical functioning**	To what extent HAE-C1-INH limits physical activities like self-care, grooming, walking, etc.	2	1
**Social role**	To what extent HAE-C1-INH interferes with normal social life and leisure activities (including sports, traveling, hobbies).	5	3
**Emotional role**	To what extent HAE-C1-INH affects emotional state/partnership/family.	3	3
**Symptoms**	Objective questions about clinical characteristics of the patient	8	0*
**Physical role**	To what extent HAE-C1-INH interferes with work, studies, or other daily activities, including a worsened performance in these activities, a limitation in the type of activities that can be performed, or difficulties in undertaking of these activities.	7	7
**Aesthetics**	To what extent the disease affects physical appearance and aesthetic habits.	2	1
**General health**	To what extent personal health assessment including current health, health perspectives for the future, resistance to disease and the influence of HAE-C1-INH on other aspects of health.	3	3
**Mental health**	To what extent general mental health, including depression, anxiety, and behavioral and emotional control are affected by HAE-C1-INH.	12	11
**Treatment**	To what extent HAE-C1-INH treatment affects daily life, regarding disease control, side-effects, availability of treatment.	11	11

* Symptoms dimension was substituted by a supplementary clinical questionnaire.

In order to assess the content and face validity of the questionnaire, the draft version was initially evaluated by a group of 8 HAE-C1-INH experts and subsequently by a group of 16 HAE-C1-INH patients, all from different regions of Spain (expert and patient rating phase). Here, the sample was also a convenience one, in which subjects were selected in order to include a representative pool of patients that was heterogeneous with regard to sex, age, level of studies, geographical origin and severity of disease. A standardized evaluation form was used by experts to assess clarity of the items, relevance for HAE-C1-INH and assignment of dimensions. The condition established for deciding to keep, reformulate or remove items was an agreement rate of 80%. Qualitative comments suggesting that items be edited or added were also taken into account. Following the expert evaluation, 21 items were removed, 1 was added, 30 were reworded, and 6 items were reassigned to another dimension. Researchers agreed to remove the clinical dimension of the questionnaire and add a supplementary specific HAE-C1-INH clinical questionnaire specially developed for this study in order to obtain objective clinical data. Another major reason for removal was to avoid the use of similar items or to eliminate ambiguous or poorly worded items. No items were deemed irrelevant. Of the 30 items that were modified, 12 were for an agreement rate of 25%, 2 for a 37.5%, 1 for a 50% and in 14 items as result of qualitative comments. The resulting 44 item version was evaluated by the patients for clarity and relevance. Their evaluation resulted in the deletion of 1 item (due to qualitative comments), the modification of 8 items (1 item with an agreement rate of 31.25%, 3 with a 25% and 4 items due to qualitative comments) and the addition of 1 item. None of the items were removed due to a lack of relevance in either evaluation. Demographic characteristics and severity scores of the patients who participated in the semi-structured interview phase and rating phase are shown in Table 6.

**Table 6:** Characteristics and Severity Score of participating HAE-C1-INH patients (convenience representative sample)

		**Semi-structured interview phase**	**Patient rating phase**
**N° patients**		45	16
**Sex**		29 women/16 men	8 women/8 men
**Age**		18-74 years old (mean age 39)	23-55 years old (mean age 37.4)
**Severity Score**	**Asymptomatic**	2	-
	**Mild**	5	4
	**Moderate**	10	5
	**Severe**	28	7

The preliminary version of the questionnaire (HAE-QOL v 1.1.) contained 44 items compiled into 9 domains. Examples of the expert and patient rating phase are shown in Tables 7 and 8.

**Table 7:** Examples of items on HAE-QoL v 1.0 and agreement rates in expert rating phase

**Item no.**	**Subscale**	Example (*)	**A (%)**	**B (%)**	**C (%)**	**D (%)**	**Final Decision**
29	Physical Role	How often has the disease prevented you from doing your work or studying properly?	100%	100%	0%	100%	Maintained
52	Symptoms	Have you been affected by not knowing how severe an episode of swelling would be?	75%	87.5%	37.5%	100%	Modified/Reassigned dimension (to Mental Health)
14	Physical functioning	How often has angioedema impeded your ability to carry out planned activities?	87.5%	87.5%	12.5%	75%	Reassigned dimension to Physical Role
56	Treatment	How often have you worried about the possibility that treatment would not be available when you needed it?	87.5%	100%	0%	100%	Maintained
13	Treatment	To what extent have you felt more at ease after having begun treatment?	62.5%	100%	50%	87.5%	Removed (QC, similar to and better wording in item 62)

A: Clarity and adequate wording – item was revised if agreement rate was below 80% or by consensus in qualitative comments. B: Relevant for HAE-C1-INH - item was removed if agreement rate was below 80% C: Need for rephrasing/modifying the item- item was reworded if agreement rate was over 20%. D: Correct domain- item was moved if agreement rate was below 80%. * These examples were translated from the Spanish to English for this paper. QC: Qualitative comments.

**Table 8:** Examples of items and agreement rates in patient rating phase

**Item no.**	**Subscale**	Example (*)	**A (%)**	**B (%)**	**Final Decision**
33	General Health	To what extent have you been prevented from using other treatments (ACE antihypertensives, oral contraception, …) due to HAE?	75%	100%	Modified (QC also taken into account)
41	Mental Health	To what extent have you felt at ease after obtaining the HAE diagnosis?	87.5%	93.75%	Removed based on QC
23	Physical Role	How often have you missed work or school/classes because of an angioedema attack?	100%	100%	Maintained
26	Aesthetics	To what extent have you been affected by the impact of secondary adverse events of attenuated androgens treatment in your physical appearance?	68.75%	100%	Modified
6	Health Conditions	To what extent have you been affected by the need to get to a health care center to receive medication for acute attacks?	81.25%	93.75%	Modified based on QC
17	Social Role	To what extent has this disease affected you in terms of taking trips or traveling?	100%	100%	Maintained

A: Clarity and adequate wording – item was revised if agreement rate was below 80% or by consensus in qualitative comments. B: Relevant for HAE-C1-INH - item was removed if agreement rate was below 80%.

In the second phase (working in progress) the preliminary version of the questionnaire presented here will undergo a complete validation process. A psychometric field study will be performed with HAE-QoL v 1.1, SF-36v2 and the specific HAE-C1-INH clinical questionnaire in a sample of HAE-C1-INH patients. Demographic data of the participating patients will also be obtained. Statistical analysis will be performed with the program SPSS/PC Program. Descriptive and psychometric analysis of the questionnaire HAE-QoL will also be performed. The psychometric study will assess data quality (including missing values), scaling assumptions (item variance, item-total correlation, multi-trait/multi-item correlation matrix), reliability (internal consistency and test-retest with calculation of Crombach’s alpha and intraclass correlation index) and evidence for validity. The association between HRQoL as measured by HAE-QoL and different factors that could affect HRQoL will be assessed using Mann–Whitney *U* test, Krushal-Wallis test or Spearman correlation, as appropriate.

## Discussion

Disease-specific measures to assess HRQoL in HAE-C1-INH patients have not been available until now. This paper describes the initial steps toward developing a disease-specific QoL questionnaire for adults with HAE-C1-INH as part of a national multi-centre research project. Such an instrument can provide a more comprehensive and accurate evaluation of the HRQoL status of HAE-C1-INH patients and insights on how the illness affects their daily life. Moreover, it could be an instrumental tool for building health policies or implementing measures to improve the health and well-being of patients with HAE-C1-INH.

The methodological approach of this study is premised on the idea that a patient´s perception of illness and related impairments is a key issue to consider when assessing HRQoL [37]. Opinions of HAE-C1-INH experts were also used as the basis for item selection. Items were modified or removed based on the responses and feedback provided by patients and experts in order to ensure content validity. Our goal was to create an instrument that patients considered thorough, respondent-friendly and useful for expanding our knowledge of the impact of HAE-C1-INH on HRQoL.

HAE-C1-INH patients and experts were recruited from different regions of Spain in an attempt to gather a variety of perspectives in a country known for its significant geographical and cultural diversity. The questionnaire addressed several dimensions of quality of life on which to base a thorough evaluation. The domain framework is provisional and represents our *theoretical model*. While the definition or scope of categories (domains) may seem to overlap, future psychometric test results will help us to decide whether to leave the categories as they are or group some of them together under a single broader domain.

Over the last few years, HRQoL has emerged as an important outcome measure of the degree of patient suffering, as well as a tool for comparing the efficacy of different treatments and assessing the results of health policy planning. However, this outcome measure is of little value if the instruments employed lack validity or reliability.

Although generic HRQoL questionnaires are readily available, they often lack the specificity necessary to adequately measure how certain aspects of a disease are related to QoL. In such cases, a disease-specific questionnaire would be preferable.

Data linking HAE-C1-INH to a burden on quality of life has been available for some time [38], however this is the first time that data has been collected using semi-structured questions. Huang et al. [25] showed that 46% of HAE-C1-INH patients were dissatisfied with the management of the disease, 85% had a constant fear of airway closure and 65% had a fear of pain. Other studies revealed that disruptive attacks caused patients to miss opportunities for career and personal development and they may have failed to reach their full potential because of high anxiety levels and depression [26-28].

In our study, the issues cited most often by both experts and patients include potentially life-threatening attacks; the adverse side effects of medication (in several cases associated with chronic treatment); the unavailability of acute specific treatment at several health care centres; hereditary transmission; the lack of a known trigger to be avoided in many cases; and the fact that it is a rare disease about which health care professionals know very little. It is especially worth noting that the relevant aspects of HAE-C1-INH were not the same for experts and patients. Aesthetics was an aspect mentioned more often by patients than by experts. On the other hand, experts were more likely to mention the adverse side effects of treatment. This finding supports the general opinion that the clinician´s view of disease severity does not necessarily match with the patient´s perception of disability. Thus, measuring HRQoL impairment could become an important part of HAE-C1-INH management. Moreover, the recent HAE-C1-INH International Working Group consensus has recommended yearly assessments be carried out using a HRQoL questionnaire [12].

There is limited data available on HRQoL in patients with HAE-C1-INH. Lumry et al. [28] administered the SF-12 to patients and found that HAE-C1-INH created a considerable humanistic burden across physical and mental health domains. HAE-C1-INH patients also had higher mean scores than population norms in the Hamilton Depression Inventory-Short form (HDI-SF). Over 42% scored >8.5, which is indicative of depressive symptomatology. A determinant factor for depression was the severity of the disease. Patients who were under long term prophylactic treatment with attenuated androgens were shown to report even lower levels of HRQoL when compared with the overall HAE-C1-INH population.

Dermatology Life Quality Index (DLQI), SF-36, and an adaptation of the Pain Disability Index have been used in HAE-C1-INH patients [26,27], but none of these questionnaires, including the SF-12, have been specifically developed or validated for HAE-C1-INH.

The HAE-QoL questionnaire is a disease-specific HRQoL measure that could be used in future research on clinical and economic matters and the identification of the determinants of HRQoL in HAE-C1-INH. This information could thereby aid health care providers and professionals in their quest to design and implement measures for improving the HRQoL of HAE-C1-INH patients.

The next step in the HAE-QoL project will be to set up a field study using the draft questionnaire (HAE-QoL v. 1.1). We will assess data quality, scaling assumption, reliability and evidences for validity.

Lastly, the final phase of this protocol will be to perform a HRQoL study with the final version of the validated questionnaire. Assessments of HRQoL can be repeated at different times in order to discover how changes in the management of the disease and changes in drug availability influence the HRQoL of HAE-C1-INH patients. We believe this is the first multi-centre research project designed to develop a specific instrument to assess HRQoL in adults with HAE-C1-INH.

## Competing interests

The authors declare that they have no competing interests that may be perceived to influence the results and discussion reported in this manuscript.

## Authors´ contributions

NP, TC and ER have made substantial contribution to initial idea and design, acquisition, analysis and interpretation of data, and have been involved in drafting and revising the manuscript. In addition, they have reviewed the manuscript for important intellectual content and have given final approval of the version to be published. CG, CL, RC, JC, AC, VC, SC, TG, MG, DH, CM, MR and MAT have made substantial contributions to the acquisition, interpretation, and analysis of data. They have provided critical reviews of the manuscript and have given final approval of the version to be published. All authors read and approved the final manuscript.

## Authors´ information

Dr. Nieves Prior, Dr. Carmen Gómez-Traseira^1^, Dr. Concepción López-Serrano^1^, Dr. Rosario Cabañas^1^, Dr. Stefan Cimbollek^5^, Dr. Teresa González-Quevedo^5^, Dr. Mar Guilarte^4^and Dr. Carmen Marcos are members of the Spanish Study Group on Bradykinin-Induced Angioedema (SGBA) (Grupo Español de Estudio del Angioedema mediado por Bradicinina: GEAB).

Dr. Teresa Caballero is an investigator from the IdiPAZ program for promoting research activities (2009), member of the Biomedical Research Network on Rare Diseases U754 (CIBERER) and coordinator of of Spanish Study Group on Bradykinin-Induced Angioedema (SGBA) (Grupo Español de Estudio del Angioedema mediado por Bradicinina: GEAB).

## Funding

This work has been partially supported by a grant from SEAIC (Spanish Society of Allergy and Clinical Immunology, Type B Grant, 2003) and FIS (Fondo de Investigaciones Sanitarias, Grant n° 060843). Dr. Teresa Caballero is an investigator from the IdiPAZ program for promoting research activities (2009).
